# Prediction of the Fundamental Period of Infilled RC Frame Structures Using Artificial Neural Networks

**DOI:** 10.1155/2016/5104907

**Published:** 2015-12-28

**Authors:** Panagiotis G. Asteris, Athanasios K. Tsaris, Liborio Cavaleri, Constantinos C. Repapis, Angeliki Papalou, Fabio Di Trapani, Dimitrios F. Karypidis

**Affiliations:** ^1^Computational Mechanics Laboratory, School of Pedagogical and Technological Education, Heraklion, 14121 Athens, Greece; ^2^Department of Civil, Environmental, Aerospace and Materials Engineering (DICAM), University of Palermo, Viale delle Scienze, 90128 Palermo, Italy; ^3^Department of Civil Engineering, Piraeus University of Applied Sciences, 250 Thivon and Petrou Ralli Street, Aigaleo, 122 44 Athens, Greece; ^4^Department of Civil Engineering, Technological Educational Institute of Western Greece, 26334 Patra, Greece

## Abstract

The fundamental period is one of the most critical parameters for the seismic design of structures. There are several literature approaches for its estimation which often conflict with each other, making their use questionable. Furthermore, the majority of these approaches do not take into account the presence of infill walls into the structure despite the fact that infill walls increase the stiffness and mass of structure leading to significant changes in the fundamental period. In the present paper, artificial neural networks (ANNs) are used to predict the fundamental period of infilled reinforced concrete (RC) structures. For the training and the validation of the ANN, a large data set is used based on a detailed investigation of the parameters that affect the fundamental period of RC structures. The comparison of the predicted values with analytical ones indicates the potential of using ANNs for the prediction of the fundamental period of infilled RC frame structures taking into account the crucial parameters that influence its value.

## 1. Introduction

The dynamic characteristics of buildings play an important role in predicting their seismic behaviour and in selecting the appropriate retrofitting approach in case of damage. One of the most significant dynamic characteristics of a building is its fundamental period. A method that can estimate the fundamental period is essential for the reliable prediction of its response to dynamic loads and it is based on the evaluation of the building's mass and stiffness. The mass can be easily determined but the assessment of the stiffness is challenging since it is influenced by several system parameters. All elements that contribute to the stiffness, including nonstructural ones, affect the vibration characteristics of a building. Thus, in order to estimate the fundamental period, several parameters must be considered including the vertical elements such as shear walls and infill panels that contribute directly to the stiffness of the building but also parameters whose influence is not as obvious. Such parameters include the structural regularity, the number of storeys and spans, the height of the buildings, the existing openings in the vertical elements, the position of loads, and the size of the members. The inclusion of all these parameters in the estimation of the vibration characteristics of a building is not trivial. Researchers and earthquake codes have provided expressions for estimating the fundamental period of a building based on the regression analysis of the periods obtained from seismic vibrations of actual buildings. These expressions provide the fundamental period as a function of the total height or the number of storeys. Their results can vary considerably especially when the comparison is between values obtained from numerical analysis with the ones obtained from measurements. Higher values are predicted from the numerical analysis since most of the times the influence of the nonstructural components or secondary parameters is not taken into account. For example, the infill panels increase considerably the stiffness of the structure affecting the fundamental period.

Previous research has shown the importance of the infill in the dynamic behaviour of the structure either experimentally [1–5] or numerically [6–16]. Nonetheless, the infill most of the times is omitted in the numerical analysis due to (i) increase of computational time, (ii) multiple effects of infill on the structural response during an earthquake: beneficial at the beginning but adverse after being damaged, (iii) lack of confidence in the behaviour of brittle materials used in the infill, (iv) influence of the construction practices, and so forth. The existence of the openings in the infill reduces their stiffness and affects the interaction with the frame, altering the overall dynamic characteristics of the structure. Several times heavy masonry infill is replaced by light partitions and vice versa influencing considerably the seismic behaviour of the structure.

Past work has shown that infill panels subjected to in-plane-loads (especially if they do not contain openings in their diagonal) can be modeled as diagonal struts of the same material connected at the corners of the frames. The diagonal struts approach is the most commonly used in modeling of the infill panels [17–20].

In the last decades, there have been many attempts to use neural networks in structural engineering [21–24]; however, to the authors' knowledge, there has been only one attempt to apply neural networks (NNs) for the prediction of the fundamental period of infilled framed structures [25]. In this study, an ANN model has been proposed with the neurons organized into input, hidden, and output layers. The input layer consists of five neurons corresponding to five input parameters (the height of building, number of bays, ratio of area of shear walls to area of floor, ratio of infilled panels to total number of panels, and type of frame). Two hidden layers, the first and second one, exist between the input and output layers consisting of six and five neurons, respectively. The Logsig transfer function and Levenberg-Marquardt Learning mechanism were used in the ANNs modeling. The output layer, composed of one neuron, provides the fundamental period.

In the present paper, the use of Back-Propagation Neural Networks (BPNNs) is proposed to predict the fundamental period of infilled framed structures considering most of the parameters affecting its value. The Back-Propagation Neural Network is based on a new heuristic algorithm that optimizes multilayer neural networks. The model is tested using a set of data obtained by analyzing a large number of infilled frames using the software FESPA and modeling the infill as diagonal struts. The results are compared with the ones obtained using various empirical expressions existing in the literature.

## 2. Building Design Codes

Different approaches have been used for estimating the fundamental period of RC frame structures with or without infill walls. The most common expression for the calculation of the fundamental period of vibration (*T*) is

(1) $$\begin{array}{c} T=C_{t}\cdot H^{3/4}, \end{array}$$

where *H* is the total height of the building (in meters) and *C*_*t*_ is a coefficient. Such an expression is derived by using Rayleigh's method assuming that the horizontal forces are linearly distributed over the building's height; the mass distribution is constant; the mode shape is linear; and the base shear is inversely proportional to *T* ^2/3^. The above expression was first adopted by ATC3-06 [26] for reinforced concrete moment-resisting frames. The European seismic design regulations [27] and the Uniform Building Code [28], among others, adopt the same expression as ATC3-06 for the evaluation of fundamental period of vibration. Building codes from different nations adopt similar expressions assigning different values to *C*_*t*_.

The UBC proposed formula has been updated in FEMA-450 [29] based on the study by Goel and Chopra [30] and the measured period of concrete moment-resisting frame buildings, monitored during California earthquakes (including the 1994 Northridge earthquake). Based on the lower bound of the data presented by Goel and Chopra [30], FEMA proposed a similar expression for RC frames that provides a conservative estimate of the base shear:

(2) $$\begin{array}{c} T=C_{r}H_{n}^{x}, \end{array}$$

where *H*_*n*_ is the height of the structure (in meters), *C*_*r*_ is equal to 0.0466, and *x* is 0.9.

The National Building Code [31] of Canada relates the fundamental period of buildings with the number of stories, *N*, above the ground, as

(3) $$\begin{array}{c} T=0.1N. \end{array}$$

Other seismic building codes including the Indian [32], the Egyptian [33], the Venezuelan [34], and the French Seismic Codes [35], in addition to the building's height *h* (in meters), take into consideration the total base dimension, *d* (in meters), of the masonry infilled RC frame. The expression for the estimation of the fundamental period of vibration from the aforementioned seismic codes is

(4) $$\begin{array}{c} T=\frac{0.09h}{\sqrt{d}}. \end{array}$$

Eurocode 8 [27], besides the general height-related expression (see (1)), provides a more accurate expression for the calculation of the coefficient *C*_*t*_, for masonry infilled reinforced concrete frames:

(5) Ct=0.075Ac,AC=∑Ai0.2+lwiH2,

where *C*_*t*_ is the correction factor for masonry infilled reinforced concrete frames, *A*_*C*_ is the combined effective area of the masonry infill in the first storey, *A*_*i*_ is the effective cross-sectional area of the wall in the direction considered in the first storey, and *l*_*wi*_ is the length of the walls in the first storey in the direction under consideration. A detailed report on the code approaches about the fundamental period of masonry infilled RC frames can be found in Morales [36], Kaushik et al. [37], and Dorji [38].

Several researchers have proposed refined semiempirical expressions for the fundamental period of RC frame structures based on the height-related formula (Table 1). In 2004, Crowley and Pinho [41] indicated the importance of developing region-specific simplified period-height formulae. Based on the assessment of 17 existing RC frames (representative of the European building stock) examined using nonlinear dynamic analyses, they proposed a period-height formula for displacement-based design. The simple relationships presented in Table 1 are valid for RC buildings without masonry infill. The examined RC frames corresponded to actual buildings from five different south European countries designed and built between 1930 and 1980 according to older design codes. Later in 2006, Crowley and Pinho [42] using eigenvalue analysis studied the elastic and yield period of existing European RC buildings of varying height. Such studies led to a simplified period-height expression for the assessment of existing RC buildings considering the presence of masonry infill at the uncracked and cracked stage.

Guler et al. [43] using ambient vibration tests and elastic numerical analyses computed the fundamental periods of RC buildings, considering the effects of infill walls. A period-height relationship was derived for a fully elastic condition.

**Figure 1:**
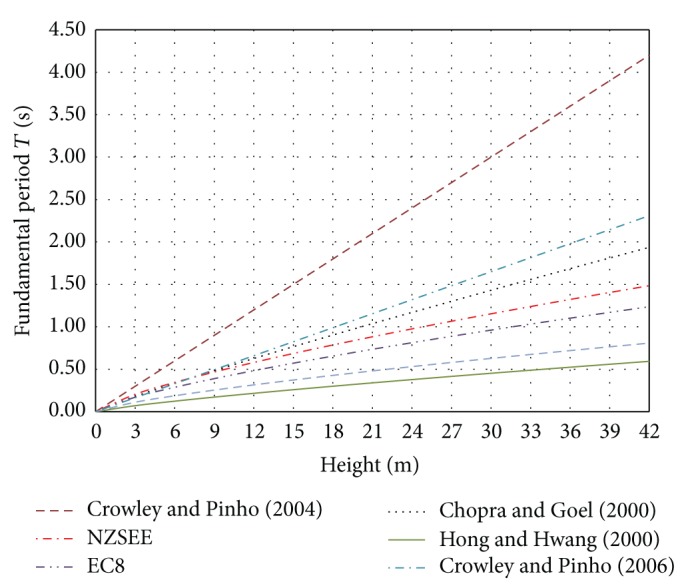
Comparison of equations for the evaluation of the fundamental period.

Figure 1 presents a comparison between some of the aforementioned height-related expressions for the evaluation of the fundamental period of vibration. It is obvious that the fundamental period calculated based on these expressions is spread out, revealing the need for further investigation and refinement of the proposals.

## 3. Description of the Database Used for Derivation of the Models

In this section, the building cases for which the fundamental period is numerically evaluated are thoroughly described. Building geometry and modeling parameters are presented. The results of the analysis will be used as input data for the neural network for the prediction of the fundamental period of any infilled frame building.

### 3.1. Building Forms and Infill Walls Parameters

**Figure 2:**
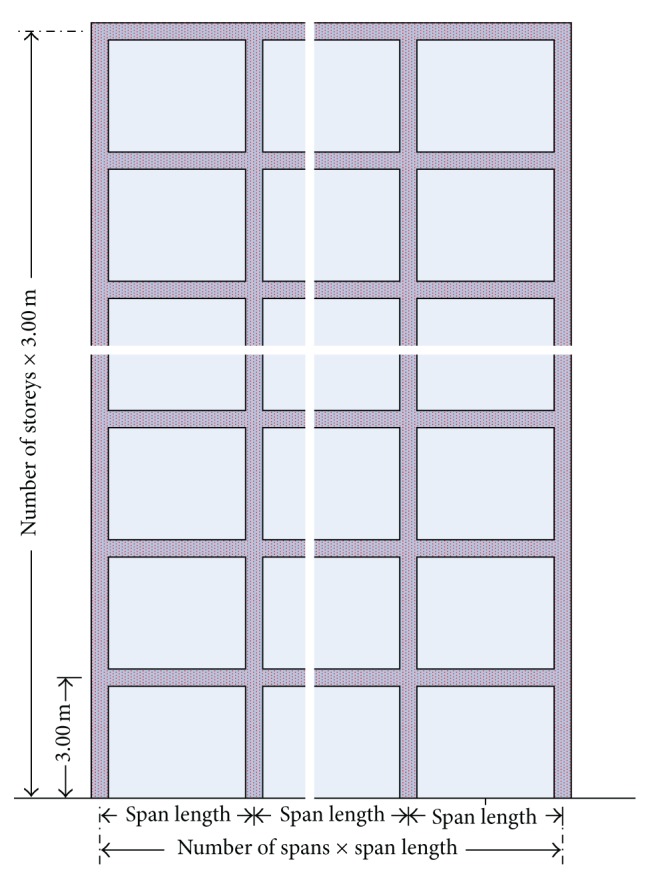
Cross section details of a RC infilled frame.

The buildings investigated in this study have 2, 4, 6, 8, 10, 12, and 14 storeys (Figure 2). The storey height for all buildings is equal to 3.0 m. The number of spans varied between 2, 4, and 6. For each case, four different span lengths (3.0 m, 4.5 m, 6.0 m, and 7.5 m) were considered.

The influence of infill walls is examined by analyzing both bare and infilled frames. Fully or partially unreinforced masonry infilled frames, with or without openings, are analyzed and various parameters are considered for each case. Infill panels are either 0.15 or 0.25 m thick, following the conventional construction of single and double leaf walls. The influence of infill wall openings is also examined by considering five different cases. Infill wall openings are given as a percentage of the panel area. Five different cases for infill wall openings are studied. These are fully infilled walls (0% openings), infill walls with small and large openings (25%, 50%, and 75% openings), and bare frames (100% openings).

Moreover, five different values for the masonry panel strength were adopted to represent weak, medium, and strong masonry, namely, 1.5 MPa, 3.0 MPa, 4.5 MPa, 8.0 MPa, and 10.0 MPa. These values cover the most common cases for masonry infill conditions in Europe.

The building parameters used for the development of the model are listed in Table 2.

The frames are designed according to Eurocode standards, using the software FESPA [44] for seismic zone I with reference peak ground acceleration on type A ground, *a*_*gR*_ = 0.16 g, for medium ductility class (DCM). The importance factor *γ*_*I*_ was set to 1.0 and the ground type B with soil factor *S* equal to 1.2 was selected, according to Eurocode 8. Square column sections were used for all frames, with low longitudinal reinforcement ratio, ranging between 1.0% and 1.5%, with most cases being under 1.15. Column dimensions range from 350 × 350 mm to 700 × 700 mm, depending on the height and the span length of the building.

### 3.2. Modeling

**Figure 3:**
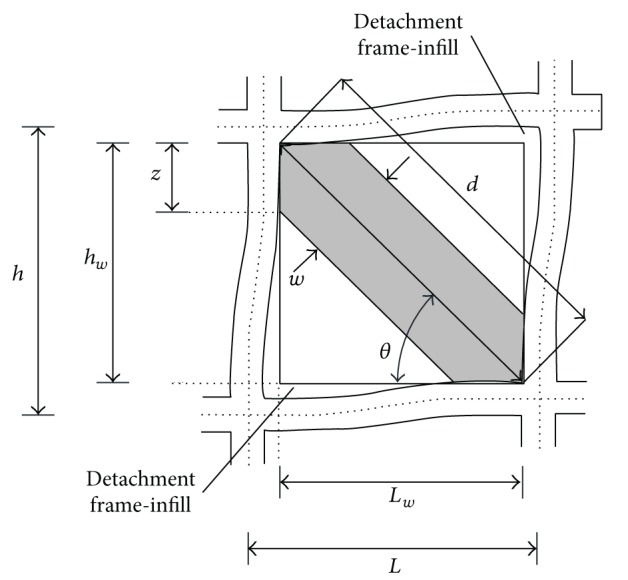
Masonry infill frame subassemblage.

Experimental and conceptual observations have indicated that a diagonal strut with appropriate geometrical and mechanical characteristics could be used to model composite infilled frame structures (Figure 3). In Figure 3, *w* is the width of the diagonal strut, *d* is the diagonal length of the masonry panel, *L* is the distance between the centres of two columns, and *z* is the contact length of the diagonal strut to the column.

Mainstone and Weeks [45] and Mainstone [46] based on experimental and analytical data evaluated the width of an equivalent diagonal strut as a function of the relative panel-to-frame-stiffness parameter using (6). FEMA-274 [47], FEMA-306 [48], and the majority of researchers adopted (6) for the analysis and rehabilitation of buildings with infilled frames

(6) wd=0.175λh−0.4,λh=hEwtwsin⁡2θ4 EIhw4,

where *E*_*w*_ is the modulus of elasticity of the masonry panel, *EI* is the flexural rigidity of the columns, *t*_*w*_ is the thickness of the infill panel and equivalent strut, *h* is the column height between centerlines of beams, *h*_*w*_ is the height of infill panel, and *θ* is the angle, whose tangent is the infill height-to-length aspect ratio:

(7) $$\begin{array}{c} \theta =\tan^{-1}\left(\;\frac{h_{w}}{L_{w}}\;\right), \end{array}$$

where *L*_*w*_ is the length of infill panel (Figure 3).

Infill wall openings are taken into account by reducing the stiffness of the infill wall. The increase in the opening percentage leads to a decrease in the frame's stiffness and for an opening percentage greater than 50% the stiffness reduction factor tends towards zero [10]. Asteris [10] proposed a stiffness reduction factor *λ* for the infill walls with openings:

(8) $$\begin{array}{c} \lambda =1-2\alpha_{w}^{0.54}+\alpha_{w}^{1.14}, \end{array}$$

where *α*_*w*_ is the ratio of the area of opening to the area of the infill wall.

The equivalent width of a strut for the case of an infill wall with opening is evaluated by multiplying the width obtained using (6) with the relevant reduction factor *λ*.

All buildings were modeled as plane frames using SeismoStruct [49]. A plastic-hinge element has been adopted for beams and columns, with concentrated inelasticity within a fixed length at each member's end. Mass was calculated using the seismic load combination, that is, dead loads plus 30% of the live loads. Masonry is modeled using the inelastic infill panel element which is an equivalent nonlinear strut, proposed by Crisafulli [50]. Design and modeling assumptions are described more thoroughly by Asteris et al. [51].

## 4. Architecture of Artificial Neural Networks

This section summarizes the artificial neural networks (ANNs) mathematical and computational aspects. Special emphasis is given on a heuristic algorithm which is proposed for the development of a reliable and robust ANN that can predict the fundamental period of RC infilled framed structures. ANNs are information processing models configured for a specific application through a training process. Trained ANN maps rapidly a given input into the desired output quantities (similar to curve fitting procedures) and thereby can be used as metamodels enhancing the computational efficiency of a numerical analysis process. This major advantage of a trained ANN over conventional numerical analysis procedures like regression analysis, under the condition that the training and validation data cover the entire range of input parameters values, is that the results can be produced with much less computational effort [52–54].

### 4.1. Back-Propagation Neural Networks

In the present study, we use a Back-Propagation Neural Network (BPNN). In this type of NNs, the output values are compared with the correct answer to compute the value of a predefined error function. By various techniques, the error is then fed back through the network. Using this information, the algorithm adjusts the weights of each connection in order to reduce the value of the error function by a small amount. After repeating this process for a sufficiently large number of training cycles, the network will usually converge to a state of small calculation error. At this stage the network has reached a certain target function. As the algorithm's name implies, the errors propagate backwards from the output nodes to the inner ones. Thus, back-propagation is used to calculate the gradient of the error of the network with respect to the network's modifiable weights. To adjust weights properly, a general method is applied for nonlinear optimization, called gradient descent. In order to minimize the error, the derivative of the error function with respect to the network weights is calculated, and the weights are then adjusted to reduce the error (thus descending on the surface of the error function). For this reason, back-propagation can only be applied on networks with differentiable activation functions. Back-propagation can be given to suitable local networks with quick convergence on satisfactory local error minima.

**Figure 4:**
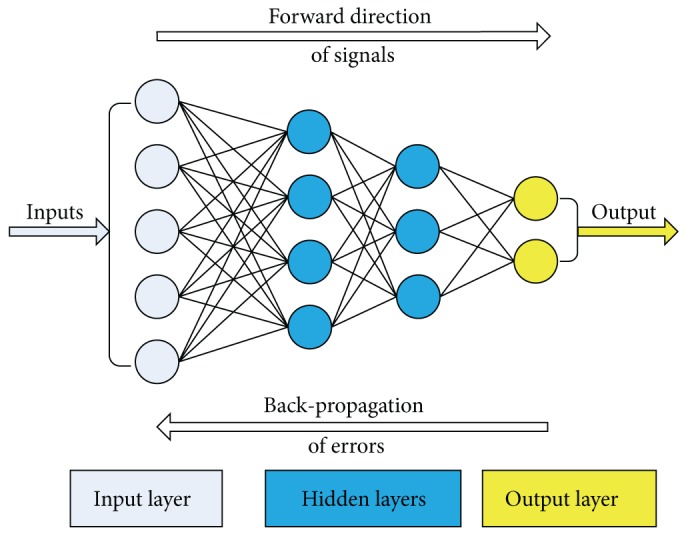
A 5-4-3-2 BPNN.

A BPNN is a feed-forward, multilayer network of standard structure; that is, neurons are not connected with each other in the layer they belong to, but they are connected with all the neurons of the previous and subsequent layer. A BPNN has a standard structure that can be written as

(9) $$\begin{array}{c} N-H_{1}-H_{2}-\cdots -H_{\text{NHL}}-M, \end{array}$$

where *N* is the number of input neurons (input parameters); *H*_*i*_ is the number of neurons in the *i*th hidden layer for *i* = 1,…, NHL; NHL is the number of hidden layers; and *M* is the number of output neurons (output parameters).  Figure 4 depicts an example of a BPNN composed of an input layer with 5 neurons, two hidden layers with 4 and 3 neurons, respectively, and an output layer with 2 neurons, that is, a 5-4-3-2 BPNN.

**Figure 5:**
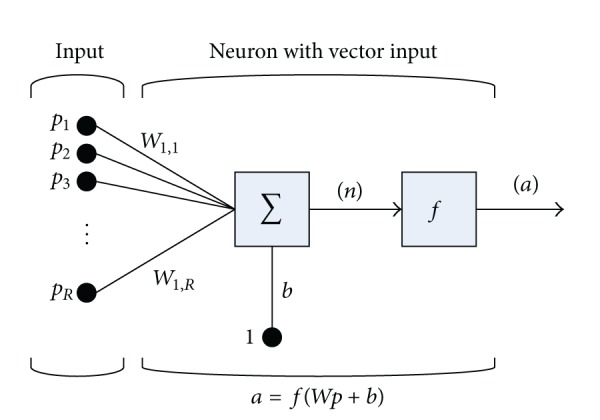
A neuron with a single *R*-element input vector.

Another notation for a single node (with the corresponding *R*-element input vector) of a hidden layer is presented in Figure 5.

For each node, the individual element inputs *p*_1_,…, *p*_*R*_ are multiplied by the weights *w*_1,1_,…, *w*_1,*R*_ and the weighted values are fed to the summing junction. At that point, the dot product (*W* · *p*) of the single row matrix *W* = [*w*_1,1_,…, *w*_1,*R*_] and the column vector *p* = [*p*_1_,…,*p*_*R*_]^*T*^ is generated. The threshold *b* (bias) is added to the dot product forming the net input *n* which is the argument of the transfer function *f*:

(10) $$\begin{array}{c} n={w_{1,1}p}_{1}+{w_{1,2}p}_{2}+\cdots +{w_{1,R}p}_{R}+b=Wp+b. \end{array}$$

### 4.2. Transfer Functions

The choice of the transfer function may strongly influence the complexity and performance of neural networks. Transfer functions are used in ANNs as activation functions connecting the weights *w*_*i*_ of a neuron *i* to the input. Although sigmoidal transfer functions are the most commonly used, there is no a priori reason why models based on such functions should always provide optimal decision borders. Past studies [55, 56] have proposed a large number of alternative transfer functions. In the present study, the following functions are used.

**Figure 6:**
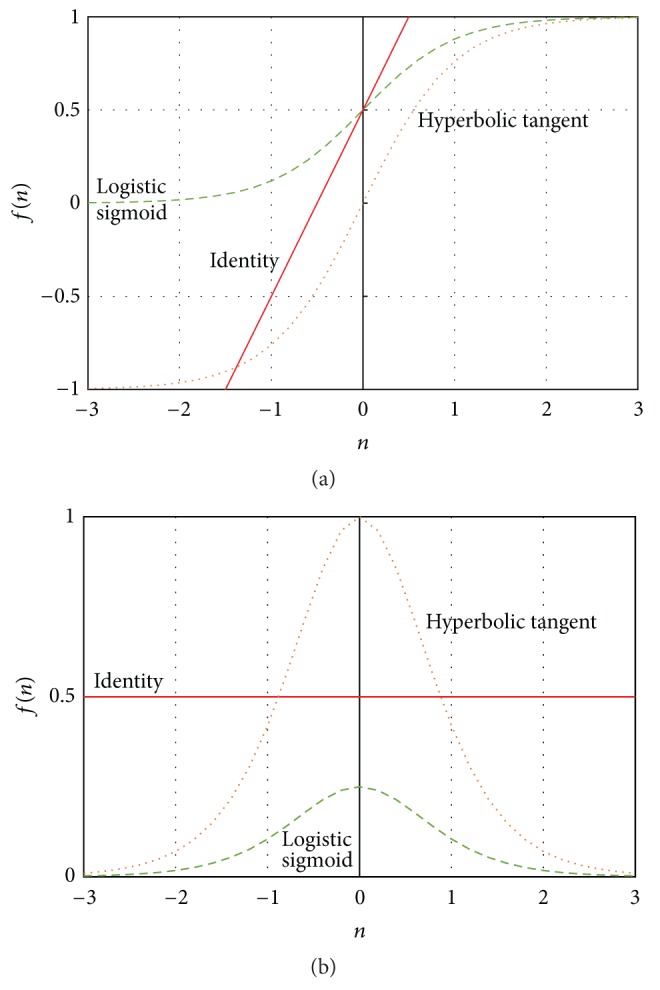
(a) Common activation functions, (b) their derivatives.

*(a) The Identity (“Linear”) Transfer Function*. The simplest transfer function commonly used is that of the identity activation function (Figure 6). The output of the identity function and its derivative are given by

(11) fn=n,f′n=1.

This function yields output values in the interval [−*∞*, +*∞*], while its derivative always yields output values equal to 1. It is worth mentioning that the combination of using nonlinear activation functions among the neurons of hidden units and the identity function for the output layer leads to a robust form of nonlinear regression. The network can predict continuous target values using a linear combination of signals that arise from one or more layers of nonlinear transformations of the input.

*(b) The Logistic Sigmoid Activation Function*. Another function, which is often used as output activation function, is the logistic sigmoid (Figure 6). The output of this function and its derivative are given by

(12) fn=1e−n+1,f′n=1+e−n+11+e−n2.

This function, yielding output values in the interval [0, +1], is suitable for binary classification problems for which the outputs values are in the interval [0, +1].

*(c) The Hyperbolic Tangent Activation Function*. An alternative to the logistic sigmoid is the hyperbolic tangent or tanh  function (Figure 6). The output of the hyperbolic tangent function and its derivative are given by

(13) fn=e2n−1e2n+1,f′n=4e2ne2n+12.

The tanh function is also sigmoidal (“s”-shaped). This function yields output values in the interval [−1, 1], while its derivative yields output values in the interval [0, 1]. Thus, strongly negative inputs to the tanh will map to negative outputs. Additionally, only zero-valued inputs are mapped to near-zero outputs. These properties make the network less likely to get “stuck” during training.

### 4.3. Finding the Best Architecture of a ANN or How to Avoid the Overfitting Problem

The best architecture of an ANN can be identified, given the known number of parameters for input and output (5 and 1, resp., for the present application), by estimating the optimum number of hidden layers and neurons.

The estimation of the best architecture avoids the overfitting problem. Overfitting generally occurs when a model is excessively complex, such as having too many parameters relative to the number of observations as well as when the training data do not cover the entire range of the input parameters values of the problem. As an extreme example, when the number of parameters is equal to or exceeds the observations, a simple model can predict the training data by memorizing them but fails to predict new ones by not learning to generalize. In order to prevent overfitting, several techniques/algorithms and criteria have been proposed [54, 57–59] for determining the correct number of neurons with their hidden layer based mainly on the number of inputs and output parameters [60–62]. In the present work, a simple heuristic algorithm is proposed achieving a reliable and robust ANN suitable for predicting the parameter/function that contains a continuous mapping from one finite space to another. The steps of the proposed algorithm that can predict the fundamental period of RC infilled frame structures are the following.

**Step 1 (development and training of several neural networks).**

The development and training of the NNs occur with a number of hidden layers ranging from 1 to *n*_ip_ − *n*_op_ and with a number of neurons ranging from *n*_ip_ − 1 to 3 × (*n*_ip_ − 1), where *n*_ip_ and *n*_op_ correspond to input and output parameters, respectively. Each one of the NNs is developed and trained for a number (*n*_*f*_) of activation functions.

**Step 2 (determination of mean square error).**

For each one of the above trained NNs, the mean square error (MSE) is computed for a set of data (validation data) which have not been used during the training process (training data) of the ANNs.

**Step 3 (establishment of upper and lower limits).**

Upper and lower limits are introduced for each one of the output parameters based on experimental or numerical data as well as reasonable estimations by the users.

**Step 4 (selection of optimum architecture).**

The optimum architecture is the one that gives the minimum mean square error while all the computed output parameters for all the validation data are between the upper and lower limits.

The importance of the limits established at Step 3 based on the user's expertise should be emphasized, since wide experience is needed not only in relation to the neural networks but also to the specific field applied in order to establish reasonable estimations.

## 5. Results and Discussion

### 5.1. Data Set

**Figure 7:**
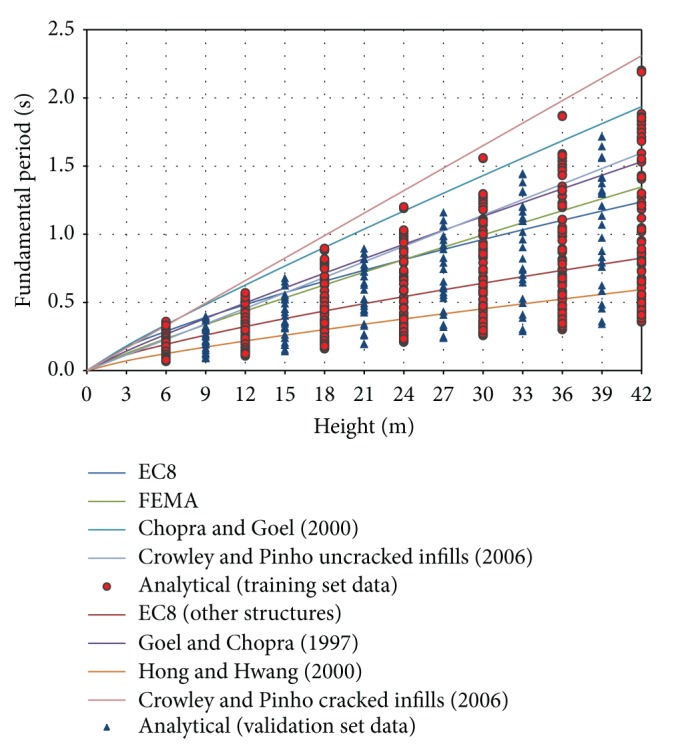
Fundamental period of infilled frame structures.

A total of 1281 infilled frames have been studied, and the fundamental period (output parameter for the NNs) was investigated. A representative range of values is used (as presented in Table 2) for the following five parameters of the model: (i) number of storeys (equivalent of the total height of structure), number of spans, span length, infill wall stiffness, and opening percentage (5 input parameters for the NNs). Due to the large size of the data sets, the results are presented graphically in Figure 7.

Figure 7 presents the numerical values of the fundamental period for the entire set of 1281 cases under study, along with the corresponding values appearing in the literature. As can be seen, there is a great discrepancy in the numerical values, and none of the up-to-date proposals yield robust estimates.

### 5.2. BPNN Model Development

Based on the algorithm described in Section 4.3, 78 BPNN models have been developed and investigated. Each one of these models was trained by means of 1053 data sets (out of the total of 1281, that is, an 82% percentage) and the reliability of the results was validated against the remaining 228 data sets (18% of total), by calculating the mean square error (MSE) using the following equation:

(14) $$\begin{array}{c} \text{MSE}=\frac{1}{\nu }\overset{\nu }{\underset{i=1}{\sum }}{\left|\;T_{i,p}-T_{i,a}\;\right|}^{2}, \end{array}$$

where *T*_*i*,*p*_ and *T*_*i*,*a*_ are the predicted and the numerical values of the fundamental period while *ν* is the number of the training data.

**Figure 8:**
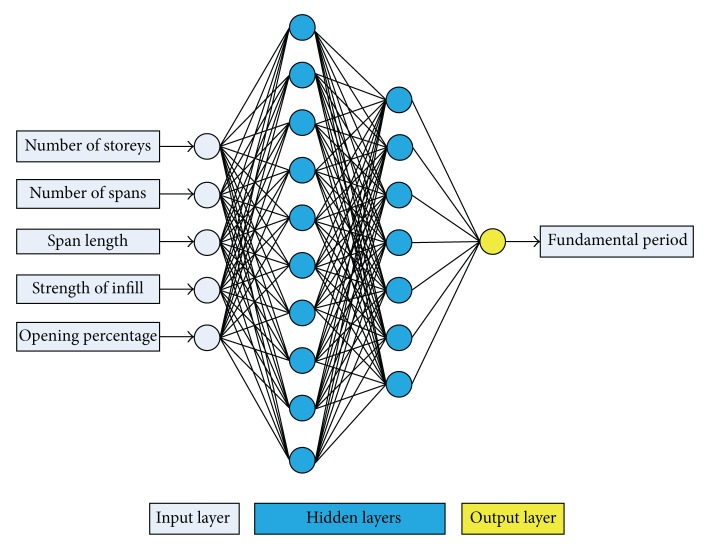
A 5-10-7-1 BPNN.

**Figure 9:**
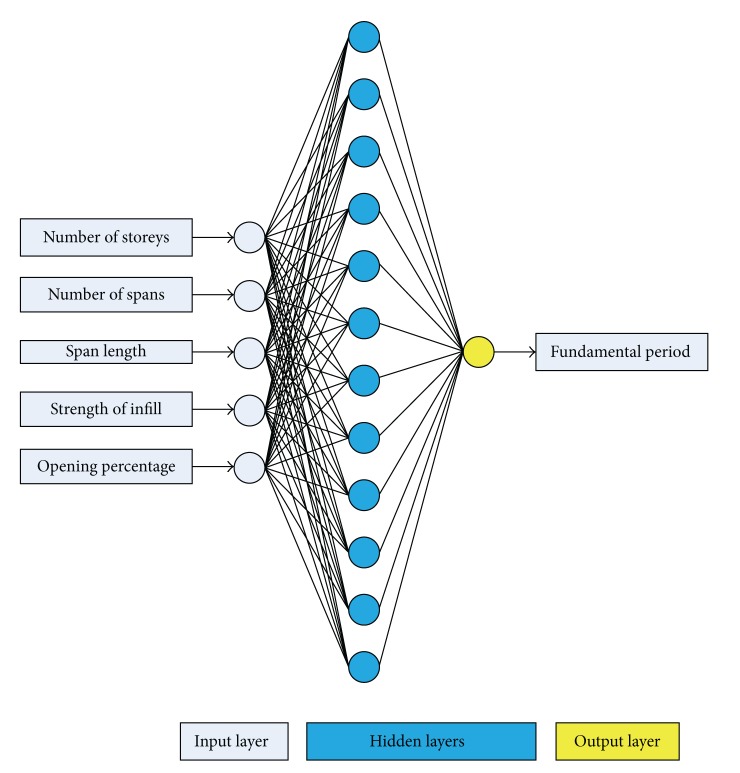
A 5-12-1 BPNN.

**Figure 10:**
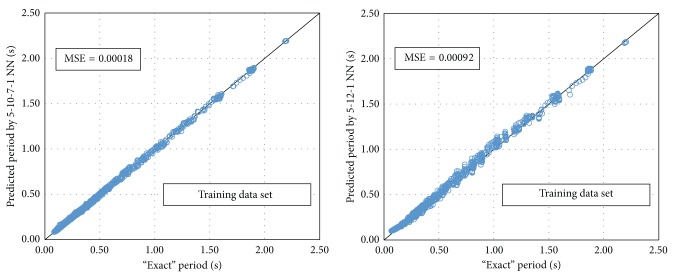
The mean square error of the “exact” and predicted fundamental period for the two NNs (training data set).

**Figure 11:**
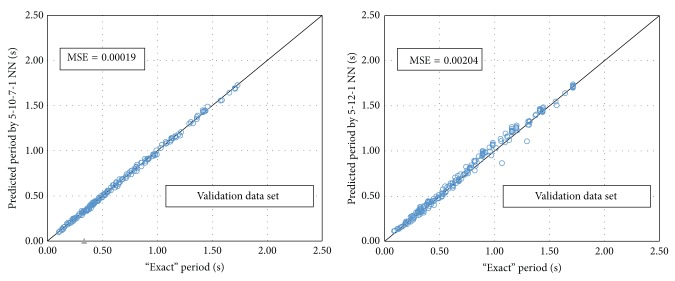
The mean square error of the “exact” and predicted fundamental period for the two NNs (validation data set).

**Figure 12:**
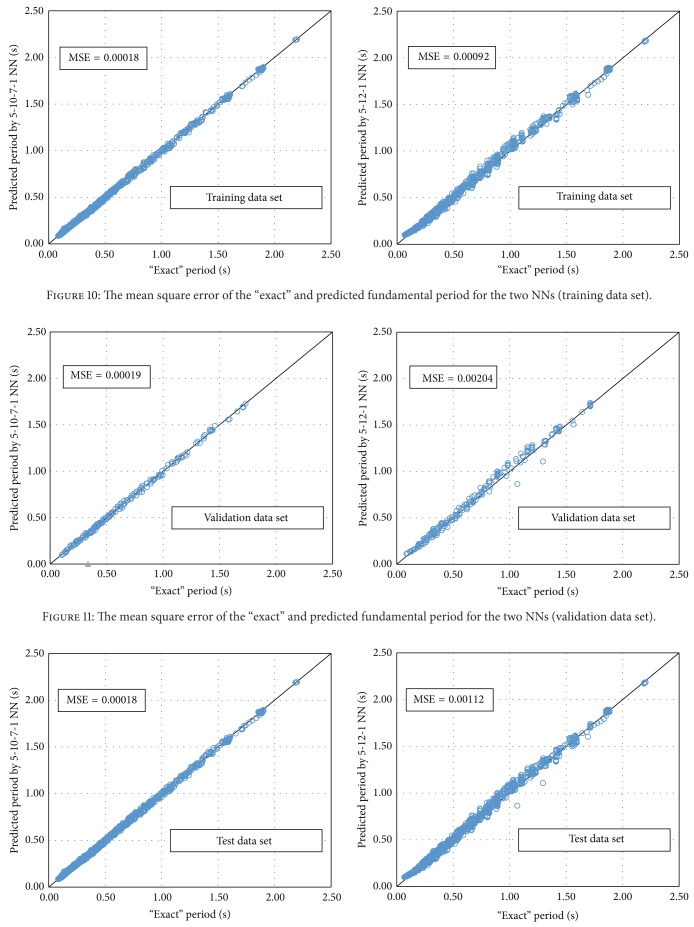
The mean square error of the “exact” and predicted fundamental period for the two NNs (test data set).

Based on this procedure, the optimum BPNN model is that of 5-10-7-1 (Figure 8) with MSE equal to 0.00019. The name of the model reveals the number of neurons used in each layer. It is worth mentioning that the best NN in regard to the computational time is the 5-12-1 (Figure 9) with MSE 0.00204. Figures 10–12 present the numerical results of the fundamental period values, as predicted by the two NNs compared to the analytically derived values. The comparison is taking place for both training data (Figure 10) and validation data (Figure 11) as well as for the test data set (Figure 12). It is also clear that the 5-10-7-1 NN with the smaller value of MSE predicts better than the 5-12-1 which has a larger MSE value with the trade of a slight increase in the computational time.

### 5.3. Comparisons with Code Provisions

**Figure 13:**
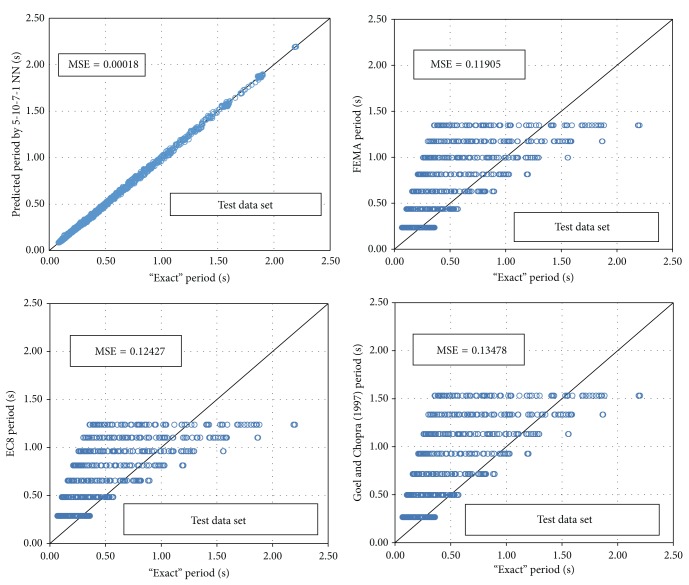
Comparison of the proposed NN with “exact” data and formulae from the literature.

The advantages of the derived BPNN model compared to the code provisions and other research formulae are shown in Figure 13. The importance of graphing the data and the effect of outliers on the statistical properties of a data set should also be noticed. For example, Anscombe [63] presented four simple data sets, and even though they show identical statistical properties (mean values, standard deviation, correlation factor, etc.), they were quite different when inspected graphically. For these reasons, Figure 13 compares the “exact” dynamic analyses results with the ones predicted by the existing empirical expressions and by the BPNN model.

From Figure 13, it is clear that the proposed 5-10-7-1 BPNN provides much more reliable values for the fundamental period of infilled frame structures than those proposed by the empirical equations, thus confirming the validity of the proposed NN. In particular, the proposed NN leads to the minimum MSE (0.00018) if compared to the other research or code proposals like FEMA-450 [29], EC8 [27], and Goel and Chopra [30] with corresponding MSE of 0.11905, 0.12427, and 0.13478, respectively. Similar observations can be made if the NN results are compared with the rest of the research proposals presented in Section 2.

## 6. Conclusions

In this paper, the artificial neural networks method was assessed by investigating its accuracy in predicting the fundamental period of infilled framed structures. In particular, a new heuristic algorithm was proposed to find the optimum structure of a set of multilayered feed-forward Back-Propagation Neural Networks. Based on this algorithm, a neural network model of two hidden layers was selected as the best fit. In the first and second hidden layers, 10 and 7 neurons were determined, respectively. The fundamental period values, predicted from the multilayer feed-forward neural network, are very close to the exact results as confirmed by the statistical parameter value MSE. Furthermore, comparison with the available code provisions has shown that the predicted periods by the BPNN model are more accurate and reliable. In conclusion, the fundamental period of infilled frame structures can be predicted by multilayer feed-forward neural network model with smaller error rates and less computational effort.

## Figures and Tables

**Table 1:** Expressions for the evaluation of fundamental period of vibration.

Expression	Reference
*T* = 0.053*H* ^0.9^	Goel and Chopra [30]
*T* = 0.0294*H* ^0.804^	Hong and Hwang [39]
*T* = 0.067*H* ^0.9^	Chopra and Goel [40]
*T* = 0.1*H*	Crowley and Pinho [41]
*T* = 0.055*H*	Crowley and Pinho [42]
*T* = 0.026*H* ^0.9^	Guler et al. [43]

**Table 2:** Building parameters.

Concrete strength	25 MPa
Modulus of elasticity of concrete, *E*_*c*_	31 GPa
Steel tensile yield strength	500 MPa
Size of beams	250/600 mm
Slab thickness	150 mm
Dead loads	1.50 kN/m^2^ + 0.90 kN/m^2^
Live loads	3.50 kN/m^2^
Number of floors	1, 2, 3, 4, 5, 6, 7, 8, 9, 10, 11, 12, 13, 14
Storey height	3.00 m
Span length	3.00 m, 4.50 m, 6.00 m, 7.50 m
Number of spans	2, 4, 6
Masonry compressive strength, *f*_*m*_	1.5 MPa, 3.0 MPa, 4.5 MPa, 8.0 MPa, 10.0 MPa
Modulus of elasticity of masonry, *E*_*m*_	1.5 GPa, 3.0 GPa, 4.5 GPa, 8.0 GPa, 10.0 GPa
Thickness of infill panel, *t*_*w*_	150 mm, 250 mm
Infill wall opening percentage	0% (fully infilled), 25%, 50%, 75%, 100% (bare frame)
